# Clinical study of Tongdu Shujin decoction in the treatment of ankylosing spondylitis with cold-dampness obstruction type: Study protocol for a randomized controlled trial

**DOI:** 10.1097/MD.0000000000032852

**Published:** 2023-02-10

**Authors:** Zihua Wang, Mingrui Du, Jigao Li, Dongliang Shi, Hejun Wang, Huan Li, Hongtao Guo

**Affiliations:** a The First Affiliated Hospital of Henan University of Chinese Medicine, Zhengzhou, China; b The Second Clinical Medical School, Henan University of Chinese Medicine, Zhengzhou, China; c Henan University of Chinese Medicine, Zhengzhou, China.

**Keywords:** ankylosing spondylitis, clinical efficacy, pain, stiffness, traditional Chinese medicine

## Abstract

Ankylosing spondylitis (AS) has a high incidence, and severe cases can lead to spinal deformity and even joint fusion, which causes a huge burden on patients life, work and psychology. Tongdu Shujin decoction (TDSJ) has a definite effect in the treatment of ankylosing spondylitis, so we designed a randomized controlled trial to observe the efficacy of TDSJ in the treatment of AS, and to evaluate its safety.

**Methods:**

In this randomized controlled trial, 80 eligible patients were randomly assigned in a 1:1 ratio to a treatment group TDSJ and a control group (celecoxib capsules in combination with thalidomide tablets) for 8 weeks. Visual analogue scale, bath ankylosing spondylitis disease activity index, bath ankylosing spondylitis functional index, and traditional Chinese medicine syndrome scores will be used as primary indicators. Erythrocyte sedimentation rate, C-reactive protein, spinal mobility (figure-ground distance, occipital tubercle-wall distance, Schober test) will be used as secondary indicators. Vital signs (respiration, heart rate, body temperature, blood pressure, electrocardiogram), blood routine, urine routine, stool routine, liver function, and renal function will be used as safety indicators. The primary and secondary indicators will be detected at 0th and 8th week, while safety indicators at 0th, 4th, and 8th week.

**Discussion:**

This study will provide high-quality clinical evidence for the efficacy and safety of TDSJ in the treatment of AS.

## 1. Introduction

Ankylosing spondylitis (AS) is a chronic inflammatory disease mainly of the sacroiliac joint and spinal erosion, patients could have joint pain, stiffness and limited range of motion, and in severe cases, spinal deformity and joint fusion. The incidence of AS in China is about 0.2% to 0.4%, the ratio of men and women is 2 to 3:1,^[1]^ the age of onset is mainly concentrated in 15 to 40 years old, and the peak time is 20 to 30 years old,^[2]^ which is a common disease and frequent incidence in Rheumatology and Immunology Department. AS inflammatory infiltration can lead to sacroiliac joint erosion, spinal adhesion calcification and even fusion, severe cases can involve peripheral joints causing hip fusion and disability, and AS hip joint involvement rate is as high as 60%.^[3]^ The high disability rate has a very negative impact on the physical, psychological and social life towards patients.

It was found that Tongdu Shujin decoction (TDSJ) had a good effect in the treatment of AS with cold-dampness obstruction type by clinical observation. Therefore, we designed this trail to evaluate the clinical efficacy and safety of TDSJ in the treatment of AS with cold-dampness obstruction type, hoping to provide high-quality clinical evidence for the treatment of ankylosing spondylitis with traditional Chinese medicine (TCM).

## 2. Methods and analysis

### 2.1. Design

**Figure 1.**
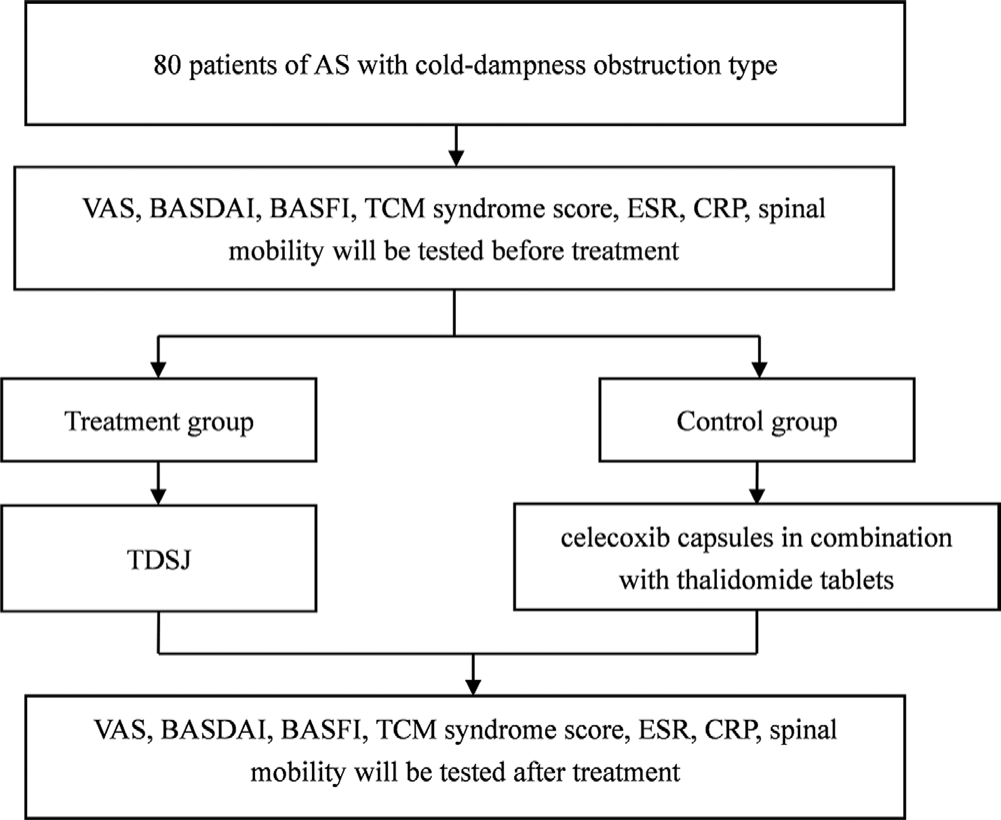
Research flow chart.

An 8-week, single-center, rater-blinded, randomized controlled trial is forthcoming. The treatment group (TDSJ interventions) and the control group (celecoxib capsules in combination with thalidomide tablets intervention) were compared to observe the efficacy of TDSJ in AS patients with cold-dampness obstruction type, and to evaluate its safety. The trial will be conducted in the Andrology Department of the First Affiliated Hospital of Henan University of Chinese Medicine. All participants will be required to provide written informed consent prior to entry into the trial. The research flow chart is shown in Figure 1.

### 2.2. Ethic approval

This protocol was carried out in accordance with the principles of the Declaration of Helsinki,^[4]^ and it had been approved by the ethics review committee of the first affiliated hospital of Henan university of Chinese medicine (approval number: 2022HL-424-01) and the whole process was followed up. We registered the study with the Chinese clinical trials registry (registration number ChiCTR2300067535).

### 2.3. Participants

After obtaining informed consent, this study will include 80 AS patients with cold-dampness obstruction type. We will post printed recruitment posters in the hospital or post recruitment information on hospital social media to recruit participants, and participants who meet the research criteria will be invited to the Rheumatology and Immunology Department of the First Affiliated Hospital of Henan University of Chinese Medicine for the research. Eligible participants will be randomized 1:1 to treatment and control groups. The treatment will last 8 weeks.

#### 2.3.1. Diagnostic criteria.

Adoption of the New York Standard revised in 1984^[5]^

Clinical criteria: The course of low back pain lasted at least 3 months, and the pain improved with activity, rest did not decrease; The movement of the lumbar spine is restricted in the anteroposterior and lateral flexion directions; The thoracic extension range is < the normal value of the same age and sex.

Radiological criteria: bilateral sacroiliitis ≥ grade II, or unilateral sacroiliitis grade III to IV.

AS is confirmed if the patient has radiological criteria and any one of the clinical criteria is appended. Diagnosis of probable AS is achieved in those who meet only 3 clinical criteria, or only those who meet radiologic criteria.

#### 2.3.2. Diagnostic criteria for TCM syndrome.

Referring to the dialectical standard of TCM on ankylosing spondylitis in the “Guidelines for Clinical Research of New Chinese Medicines”^[6]^ issued by the National Medical Products Administration, it is proved to be cold-dampness obstruction type.

Primary symptoms: lumbosacral pain, back pain, limited lumbar spinal movement, morning stiffness, fever, redness, swelling and heat pain in the joints of the limbs, swelling and pain in the eyes.

Secondary symptoms: thirst or dry mouth, heavy limbs, dry stools, yellow urine.

Tongue veins: red tongue, moss yellow or yellow thick, smooth pulse.

#### 2.3.3. Inclusion criteria.

The inclusion criteria are as follows: Meet the Western medical diagnostic standards of AS; TCM dialectic is cold-dampness obstruction type; 18 to 40 years old, male or female; Women of childbearing age ascertain to be non-pregnant and had no pregnancy plans for the duration of the trial; Did not take any AS treatment drugs in the 1 month period before receiving treatment; Voluntarily participate in this trial and agree to enter the clinical study, sign the informed consent form.

#### 2.3.4. Exclusion criteria.

The exclusion criteria are as follows: Patients with other rheumatic immune system diseases; Those with serious diseases such as heart, brain, lung, liver, kidney, tumor, infection or hematopoietic system; Patients who are taking other Chinese medicine treatment for certain diseases; Lactating or pregnant women; Those who are allergic to the drugs in this test; Patients with mental disorders who cannot communicate effectively; Active peptic ulcer/bleeding, sulfa allergy; Patients with severe heart failure; Patients with perioperative pain during coronary artery bypass graft.

### 2.4. Sample size

The sample size calculation was performed using PASS 15 software (https://www.ncss.com/),^[7]^ with α = 0.05 (2-tailed test). The sample size was N_1_ in the treatment group and N_2_ in the control group. According to the current clinical efficacy statistics of TCM in the treatment of AS, the effective rate of the treatment group was P_1_ = 78%, while the control group P_2_ = 43%. Using the Z test, H_0_: P_1_–P_2_ = 0. H_1_: P_1_–P_2_ = D_1_ ≠ 0, calculated using the approximate normal method, N_1_ = N_2_ = 36. Considering the loss of cases, if the loss rate is 10%, and each group increases by 4 people, the final sample size is 40 cases in the treatment group and the control group, for a total of 80 cases.

### 2.5. Randomization and blinding

This trail will use random assignment concealment. Participants who provide informed consent and meet the inclusion criteria will be randomly assigned in a 1:1 ratio to treatment and control groups. Randomization will be based on random numbers generated by the computer. Independent researchers will prepare the distribution in an opaque envelope containing the distribution sequence number and are responsible for concealing the distribution sequence. Due to the specificity of the intervention, double-blinding was impossible. Outcome assessors, data curators will be unaware of treatment assignments.

## 3. Interventions

### 3.1. Basic interventions

Patients are advised to eat a light diet, quit smoking and alcohol, keep warm appropriately, maintain a comfortable mood, and avoid overwork during the treatment.

### 3.2. Drug intervention

#### 3.2.1. Treatment group.

Patients in this group will take TDSJ orally. The composition of TDSJ are as follows: astragalus 30g, coix seed 30g, pueraria lobata 30g, dipsacus asperoides 20g, taxilli herba 20g, rhizoma drynariae 20g, eucommia 20g, rehmannia glutinosa 15g, pyrola 15g, paeoniae alba 15g, achyranthes bidentata 20g, ground beetle 10g, ligusticum chuanxiong 20g, angelica sinensis 15g, safflower 10g, clematis chinensis 20g, angelicae pubescentis radix 10g, corydalis yanhusuo 20g, licorice 6g. The drug will be uniformly provided by the Chinese Pharmacy of the First Affiliated Hospital of Henan University of Chinese Medicine, decocted in water, 400mL per payment, 1 payment per day, divided in the morning and evening, oral for 8 weeks.

#### 3.2.2. Control group.

Patients in this group will take celecoxib capsules in combination with thalidomide tablets. Celecoxib capsules are provided by Sichuan Guowei Pharmaceutical Co., Ltd., Sinopharm H20203357, specification 0.2g*20 capsules, 1 capsule in the morning and evening, oral for 8 weeks; Thalidomide tablets are provided by Changzhou Pharmaceutical Factory Co., Ltd., Sinopharm H32026129, specification 50mg*20 tablets, 2 tablets in the evening, oral for 8 weeks.

### 3.3. Combined treatment regulations

If the subjects meet the inclusion criteria, and the patients with other diseases need to continue to use the drug in the clinical trial, or if they really need to add other drugs or treatment methods due to the needs of the disease treatment, the drugs used should be recorded in the case report form in detail. name (or treatment method), dosage, frequency of administration, time of administration, etc. The drugs and treatment methods that must be taken in combination with other diseases must be recorded in detail in the combined medication table. If disease progresses during the study, participants can withdraw from the study and use other treatments. This case will be treated as an excluded case and the patient will be asked to complete relevant examinations and assessments as far as possible.

## 4. Outcome measures

### 4.1. Primary outcome

Visual analogue scale, bath ankylosing spondylitis disease activity index, bath ankylosing spondylitis functional index, and TCM syndrome scores will be primary indicators, which will be tested at 0th and 8th weeks of treatment.

### 4.2. Secondary outcomes

Erythrocyte sedimentation rate, C-reactive protein, spinal mobility (figure-ground distance, occipital tubercle-wall distance, Schober test) will be secondary indicators, which will be tested at 0th and 8th weeks of treatment.

### 4.3. Safety outcomes

Vital signs (respiration, heart rate, body temperature, blood pressure, electrocardiogram), blood routine, urine routine, stool routine, liver function, and renal function will be safety indicators, which will be tested at 0, 4, and 8 weeks of treatment. If an adverse event occurs, the clinical investigator will record it in detail on the case report form (including symptoms, time of onset, duration, examination and results). Serious adverse reactions will be reported to the Ethics Committee of the First Affiliated Hospital of Henan University of Chinese Medicine and rescue procedures will be initiated immediately.

### 4.4. Quality control and trial monitoring

Before the trial begins, all investigators will receive specialized training to ensure the quality of the study. Training includes how to select and exclude participants, how to complete randomization, how to properly implement interventions, how to record case report forms in a standard way, how to evaluate outcomes and manage data. Clinical investigators responsible for diagnosis and treatment will be registered Chinese medicine practitioners. In order to improve the compliance of the participants, the researchers will conduct health education and fully respect the participants right to informed consent. Raw data will be recorded in a case report form, and 2 data administrators will enter the data into the spreadsheet and review the data separately. To ensure the objectivity of the data, the evaluation and statistics during the trial are blinded. The lead investigator will oversee the entire trial.

### 4.5. Statistical analysis

SPSS 28.0 software (https://www.ibm.com/spss) will be used by professionals blinded to trial grouping for statistical analysis of data. Efficacy and safety analyses will be based on the intent-to-treat principle of analysis of all randomized participants. Measurement data that conform to normal distribution are represented by mean and standard deviation (‾ *x ± s*), and measurement data that are not normally distributed are represented by median and quartile (M[Q _25_, Q _75_]). Qualitative data is expressed as a %. If the data obey the normal distribution and the variance is homogeneous, the *t* test would be used for comparison; if the data does not conform to the normal distribution or the variance is unequal, the nonparametric test should be used for comparison. A 2-tailed test will be used, and *P* < .05 indicated a statistically significant difference.

## 5. Discussion

AS is a serious heterogeneous disease, mostly chronic, progressive development, long course and easy to recur, causing great burden to the patient body and psychology. Its pathogenesis is not clear yet, mostly believed to be related to heredity,^[8]^ immune disorders,^[9]^ etc. The primary goal of treatment is to control inflammation, prevent joint damage, and improve the quality of life of patients. Modern medical treatment methods mainly include non-steroidal anti-inflammatory drugs, disease-modifying antirheumatic drugs, tumor necrosis factor inhibitor, etc.^[10]^ However, there are limitations such as gastrointestinal damage and high cost. Traditional Chinese medicine has a long history of treating AS, which can compensate for these limitations to a certain extent and help improve the quality of life of patients.

AS with cold-dampness obstruction type is caused by cold and humidity leading to blood coagulation, causing muscle and joint stiffness and pain. TDSJ has the effect of warming, dispelling dampness, channeling and relieving pain. AS is mainly manifested as an inflammatory response accompanied by bone destruction, microglia are activated to produce a variety of pro-inflammatory cytokines combined with neurons, neuronal excitability is enhanced and pain occurs. Modern pharmacology shows that most of the drugs in TDSJ have anti-inflammatory and analgesic effects. Astragaloside IV in astragalus inhibits the secretion of cytokines and the expression of inflammation-related genes,^[11]^ and inhibits the development of inflammation by regulating the MAPK and STAT pathways.^[12,13]^ Coix seed extract reduces pro-inflammatory factor levels and COX-2 expression.^[14]^ Puerarin, the active component of pueraria lobata, can significantly inhibit the expression of mRNA of inflammatory mediators interleukin-1β (IL-1β), interleukin-6 (IL-6), and tumor necrosis factor-α, and significantly improve inflammatory pain.^[15]^ Asperosaponin VI, the component contained in the dipsacus asperoides could significantly inhibit RANKL-induced osteoclast formation and bone resorption activity by suppressing the formation of actin ring. It also inhibited the expression of various osteoblastic marker genes and signaling pathways, and can improve bone destruction caused by inflammation.^[16]^ Taxilli herba extracts significantly reduce levels of pro-inflammatory factors and inhibit inflammatory responses.^[17–19]^ Naringin is the main active substance of rhizoma drynariae, which can boost autophagic flux through SIRT1 upregulation via AMPK activation, thus protecting cells against inflammatory response, oxidative stress, and impaired cellular homeostasis.^[20]^ Eucommia extract can reduce serum IL-6 and IL-17 levels and relieve inflammation.^[21]^ The anti-inflammatory effect of rehmannia glutinosa is also particularly obvious, and its active ingredient catalpol can significantly decreased the expression of NF-*κ*B/NLRP3 inflammatory axis and inflammatory factors, inhibit tissue inflammation and relieve pain.^[22]^ Meanwhile, its active ingredient aucubin also has anti-inflammatory and antioxidant pharmacological effects.^[23]^ Pyrola extract inhibits phosphorylation of p38 MAPK and NF-*κ*B, inhibits the expression of inducible nitric oxide synthase inducible nitric oxide synthase, iNOS and exerts anti-inflammatory effects.^[24]^ Paeoniae alba has obvious analgesic, anti-inflammatory and immune regulating effects, and its active ingredient paeoniflorin can downregulate the NLRP3 inflammasome pathway to reduce inflammation,^[25]^ has a 2-way regulatory effect on the value-added of immune cells, and can reduce the production and release of prostaglandin E2 in the cerebral cortex,^[26]^ and is often used clinically to treat and relieve various types of pain and immune disorders. Achyranthes bidentata saponin has been shown to have osteoprotective effects on the inflammatory response induced by IL-1β stimulation, improving cellular inflammation and apoptosis.^[27]^ Chuanxiong and angelica extracts exert anti-inflammatory effects by hindering inflammatory signaling.^[28,29]^ The active ingredient of Safflower, safflower yellow, has anti-inflammatory, analgesic, and immune regulation functions.^[30]^ Both clematis chinensis and angelicae pubescentis radix extract exert anti-inflammatory effects by reducing pro-inflammatory cytokine levels.^[31,32]^ Corydecumine G (Cor G), the active ingredient of corydilis yanhusuo, has the effect of inhibiting neuronal excitation, thereby relieving pain symptoms.^[33]^ Licorice extract can inhibit the activation of a variety of inflammasome and downregulate NF-*κ*B expression, thereby reducing oxidative stress, inflammation, and pain response.^[34]^

AS has a long course and lacks definitive and effective treatment so far. As the disease progresses, it can involve the axial joint to the peripheral joint, progressively worsen, and even produce joint fusion and lifelong disability, which has a great impact on the patient and the family. Therefore, it is of great significance to find safe and effective drugs for the treatment of AS.

A limitation of this trial is that due to the nature of the intervention, double-blinding was not possible. Therefore, we will do our best to ensure that outcome assessors and data managers are unknown about the allocation scheme. The inclusion and exclusion criteria were strictly followed to improve the homogeneity of subjects. We hope that this study will provide high-quality clinical evidence for observing the efficacy and safety of TDSJ in the treatment of AS.

## Trial status

Patient enrollment will begin on February 1, 2023, and the required sample size is expected to be reached by August 31, 2023. The agreement was submitted prior to the completion of recruitment.

## Acknowledgements

We would like to thank all the patients who will participate in the trial and the staff for their support.

## Author contributions

**Conceptualization:** Zihua Wang, Hongtao Guo.

**Investigation:** Mingrui Du, Jigao Li.

**Project administration:** Dongliang Shi, Hejun Wang.

**Writing – original draft:** Zihua Wang.

**Writing – review & editing:** Huan Li.
